# Vestiges of the Natural History of Creation

**Published:** 1853

**Authors:** 


ART. XV.—*Vestiges of the Natural History of Creation.* Tenth Edition. London: 1853.

WE wonder much to see that new editions of this work are still called for by the reading public of England. "Time was, that when the brains wore out, the man would die;" and certainly if this book has not been decapitated and buried, we are not judges of a critical execution. We had supposed that after life's fitful fever it was sleeping well. It was extremely active during its brief career in this country, but for some years past it has seldom been heard of, except as a philosophical romance which dazzled the multitude for a time but now was credited by no one. It has fallen into disrepute and oblivion on this side of the Atlantic. But its fate appears to be different on its native soil. Ten editions have been consumed by the British readers of such pseudo philosophy.

This writer, with a smattering of science, took up the old notion of Lord Monboddo, which has found later advocates in Lamarck and Geoffrey St. Hiliaire, and popularized it in the "Vestiges." He would persuade the common people, as these savans would have had philosophers believe, that creation has proceeded upon a plan of development,—the lowest animals formed from slime, and the higher developed out of these, up to the highest. Upon this hypothesis, monkeys are the progenitors of men, as oysters are the stock from which monkeys sprang! The theory has been a great favorite with philosophers of a particular school, but unfortunately it is opposed by the facts of science, and it is only closet philosophers who have embraced it. It rests upon false assumptions, and the few appearances which once gave a sort of plausibility to it are giving way to the increasing light of zoological science. There has not been the progress in created beings that the theory assumes as its starting point. All the classes of the animal kingdom, if they did not start together, were originated when the earliest deposits were taking place, and the first species of each class were as perfect in organization, as high in the animal scale, as those which have succeeded them, and are living at the present day.

But we will not waste words on a book which, to all intents and purposes, may be regarded as dead, at least in all the region where our Journal circulates.

